# Metastatic Castrate-Resistant Prostate Cancer: A Rare Cause of Bowel Obstruction

**DOI:** 10.7759/cureus.20642

**Published:** 2021-12-23

**Authors:** Syed Ehsanullah, Syeda Zarmeena Rashid, Saiyed Abdullah A Ehsanullah

**Affiliations:** 1 Medicine, Washington University School of Medicine, Saint Louis, USA; 2 Internal Medicine, Dow University of Health Sciences, Dow International Medical College, Karachi, PAK; 3 Internal Medicine, Ziauddin University, Karachi, PAK

**Keywords:** prostrate cancer, castrate resistant prostrate cancer, malignant bowel obstruction, bowel obstruction, adenocarcinoma prostrate

## Abstract

Prostate cancer most commonly metastasizes to bone, lymph nodes, lungs, or liver, but rarely spreads to the large intestine. This case highlights a rare case of castrate-resistant prostate cancer (CRPC) that spread locally to the large intestine and rectum, significant enough to cause bowel obstruction. Metastatic prostate carcinomas are considered an infrequent cause of bowel obstruction.

## Introduction

Prostate cancer is the most common cancer among men after dermatological cancers. It is also one of the leading causes of cancer deaths among men of all races [1]. Several risk factors have been linked to prostate adenocarcinoma, including a family history of the disease, ethnicity, and age (>50 years) [1,2]. Prostate cancer is a slow-growing cancer with a 5-year and 10-year survival rate of >90% [1]. Prostate cancer treatment includes surveillance, radiation, surgery, chemotherapy, hormonal therapy, and immunotherapy. Castrate-resistant prostate cancer (CRPC) is a type of advanced prostate cancer that is independent, no longer responds to testosterone lower therapy. Prostate cancer usually spreads through the lymphatic system but rarely advances locally to nearby structures [3].

## Case presentation

A 59-year-old male was presented to the ED with a history of CRPC, Gleason score 5+5 = 10. When he was admitted to the hospital in January 2020 for a 30-pound weight loss and back pain, he was diagnosed with CRPC. Ct-scan demonstrated an enlarged prostate, retroperitoneal lymphadenopathy, and diffuse osseous involvement. Lymph node biopsy confirmed the diagnosis of prostatic adenocarcinoma. He was initially started on Degarelixin when his prostate-specific antigen (PSA) was 37.23, followed by Leuprolide four weeks later when his PSA was 8.0. He was later started on Abiraterone and Prednisone for 11 months. Later, with continued disease progression, Abiraterone was stopped when PSA increased to 183.70. Next, a total of six cycles of Docetaxel were given. The PSA was 183.70, 207.10, 262.30, 252.90, and 346.20 for each cycle, respectively. The subsequent line of treatment was two cycles of carboplatin + Cabizitaxel, which was discontinued due to disease progression and increased PSA to 392.0 and 480.3 with respective cycles. The patient is currently on enzalutamide with the most recent PSA of 273.50.

The patient’s symptoms have progressively worsened in the last three weeks. He presented to the hospital with diffuse abdominal pain and diarrhea. A CT scan in the ED demonstrated dilation of the colon, circumferentially involved by infiltrative soft tissue, raising the suspicion of partial or impeding large bowel obstruction secondary to malignant involvement. Flexible sigmoidoscopy revealed an area 15-20 cm from the anal canal appeared narrowed due to a lack of distensibility, but a mechanical stricture was not appreciated. Large intestine and rectum biopsy samples obtained during the Flex sigmoidoscopy were consistent with prostatic adenocarcinoma. The immunostain panel was positive for NKX3.1 while negative for CK7 and CK20.

**Figure 1:**
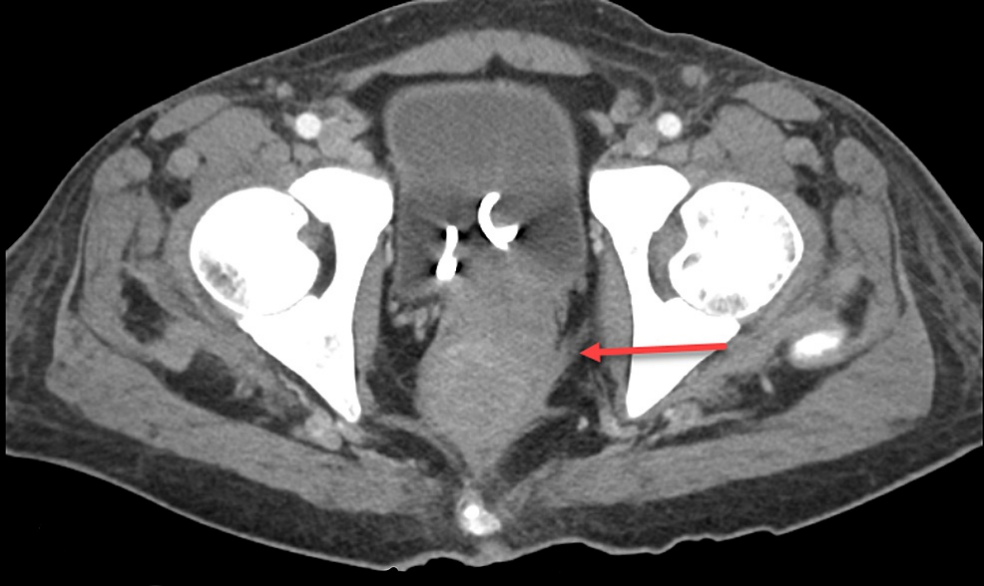
CT cross-sectional view: red arrow demonstrates infiltrative soft tissue leading to large bowel and rectum obstruction.

**Figure 2:**
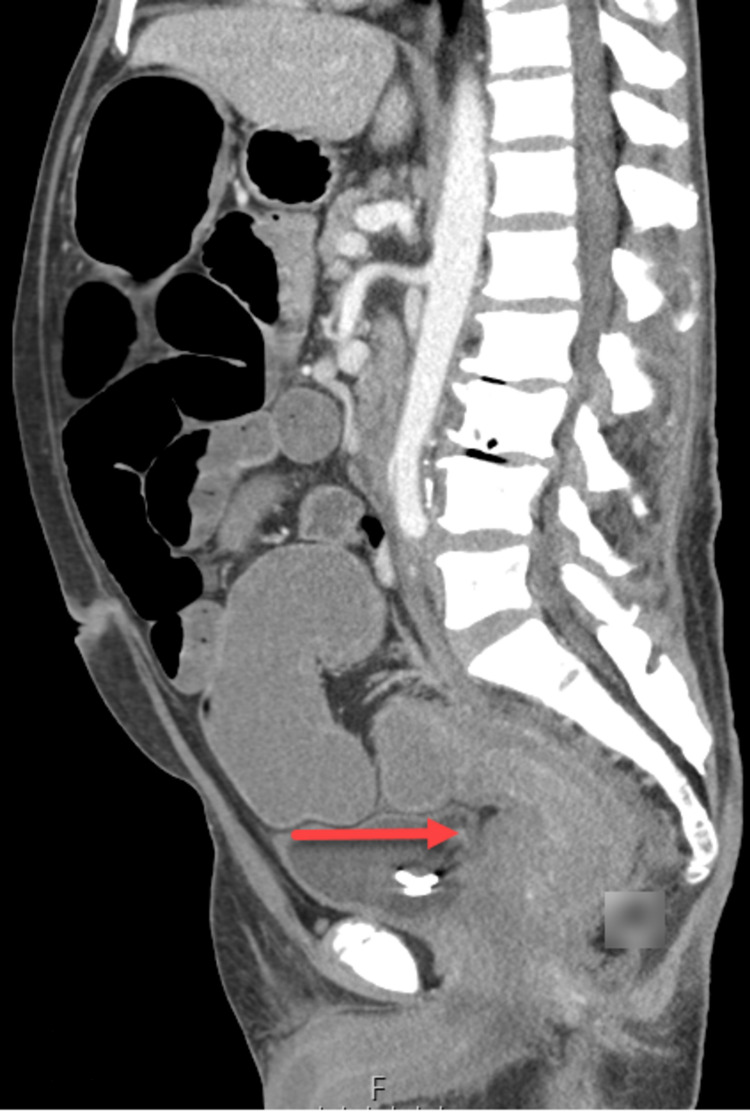
CT sagittal view: red arrow demonstrates infiltrative soft tissue leading to large bowel and rectum obstruction.

During this hospitalization, his abdominal pain worsened, and a repeat CT scan four days later showed progression of the previously demonstrated findings, now with both colonic and small bowel obstruction (Figures 1, 2). Colorectal surgery was later planned for a diverting colostomy. A successful loop descending colostomy was performed, and the patient was subsequently discharged with outpatient surgery and oncology follow-up. 

## Discussion

Prostate cancer is a slow-growing carcinoma. Early signs and symptoms include difficulty urinating, including pain or trouble starting and stopping during urination. It is easily missed during the early stages, and patients present with complications when the disease has metastasized to lymph nodes and bones [4].

Prostate cancer primarily spreads through the lymphatic system into lymph nodes, from where it spreads to the bones, liver, lungs, and rarely to the brain. There is less commonly a direct invasion through the Denonvilliers facia and into the rectum [3]. In extremely rare circumstances, it can also be an iatrogenic spread as a complication of needle biopsy if the needle penetrates the rectum [5,6].

Bowel obstruction is commonly seen in carcinomas like colon, stomach, ovary, lung, breast, and melanoma. It is rare to have bowel obstruction secondary to malignant involvement of CRPC as prostate cancer is a slow-growing carcinoma. Rectum invasion by prostate carcinoma is an indicator of late-stage prostate adenocarcinoma, and 38% of these patients survive beyond five years [7,8].

Androgen deprivation therapy (ADT) is the primary systemic therapy for regional or advanced prostate carcinoma. It can be given as monotherapy as well as neoadjuvant/adjuvant/concomitant therapy in combination with surgical castration and radiation [9]. With ADT, the target is to keep serum testosterone levels <50 ng/dL because low nadir serum testosterone levels were shown to be associated with improved cause-specific survival in the PR-7 study [10]. For chemical ADT, we typically use a luteinizing hormone-releasing hormone (LHRH) agonist or antagonist, or an LHRH agonist plus a first-generation antiandrogen like Abiraterone. Chemotherapy like Docetaxel and Cabazitaxel are also options available that can be used as adjuvants or neoadjuvants to ADT [10]. Pembrolizumab is an anti-PD1 antibody that is used for patients with unresectable or metastatic tumors who have progressed on prior treatment and who have no satisfactory alternative treatment options [11].

## Conclusions

Castrate-resistant prostate adenocarcinoma rarely invades the rectum and large intestine. It is also quite rare for prostate cancer to grow to a size that causes significant bowel obstruction. All patients with bowel obstruction due to a new infiltrative intestinal mass should be biopsied, even if they have a known cancer. Some rare cancers, like the prostate, can also infiltrate the intestine and rectum.

## References

[1] (2021). National Cancer Institute Physician Data Query (PDQ) Prostate Cancer Prevention. https://www.cancer.gov/types/prostate/hp/prostate-prevention-pdq.

[2] Siegel RL, Miller KD, Fuchs HE, Jemal A (2021). Cancer statistics, 2021. CA Cancer J Clin.

[3] Tang T, Yang Z, Zhang D, Qu J, Liu G, Zhang S (2017). Clinicopathological study of 9 cases of prostate cancer involving the rectal wall. Diagn Pathol.

[4] Hematpour K, Bennett CJ, Rogers D, Head CS (2006). Supraclavicular lymph node: incidence of unsuspected metastatic prostate cancer. Eur Arch Otorhinolaryngol.

[5] Lane Z, Epstein JI, Ayub S, Netto GJ (2008). Prostatic adenocarcinoma in colorectal biopsy: clinical and pathologic features. Hum Pathol.

[6] Vaghefi H, Magi-Galluzzi C, Klein EA (2005). Local recurrence of prostate cancer in rectal submucosa after transrectal needle biopsy and radical prostatectomy. Urology.

[7] Abbas TO, Al-Naimi AR, Yakoob RA, Al-Bozom IA, Alobaidly AM (2011). Prostate cancer metastases to the rectum: a case report. World J Surg Oncol.

[8] Wang H, Yao Y, Li B (2014). Factors associated with the survival of prostate cancer patients with rectal involvement. Diagn Pathol.

[9] Mohler JL, Antonarakis ES, Armstrong AJ (2019). Prostate cancer, version 2.2019, NCCN Clinical Practice Guidelines in Oncology. J Natl Compr Canc Netw.

[10] Klotz L, O'Callaghan C, Ding K (2015). Nadir testosterone within first year of androgen-deprivation therapy (ADT) predicts for time to castration-resistant progression: a secondary analysis of the PR-7 trial of intermittent versus continuous ADT. J Clin Oncol.

[11] (2018). KEYTRUDA® (pembrolizumab) for injection, for intravenous use
KEYTRUDA® (pembrolizumab) injection, for intravenous use
Initial U.S. Approval: 2014. NJ.

